# Prevalence of Positive Effects on Body Fat Percentage, Cardiovascular Parameters, and Cardiorespiratory Fitness after 10-Week High-Intensity Interval Training in Adolescents

**DOI:** 10.3390/biology11030424

**Published:** 2022-03-10

**Authors:** Jarosław Domaradzki, Dawid Koźlenia, Marek Popowczak

**Affiliations:** 1 Unit of Biostructure, Faculty of Physical Education and Sport, Wroclaw University of Health and Sport Sciences, al. I.J. Paderewskiego 35, 51-612 Wroclaw, Poland; jaroslaw.domaradzki@awf.wroc.pl; 2 Unit of Team Sports Games, Faculty of Physical Education and Sport, Wroclaw University of Health and Sport Sciences, al. I.J. Paderewskiego 35, 51-612 Wroclaw, Poland; marek.popowczak@awf.wroc.pl

**Keywords:** adolescent, Tabata training, high-intensity interval training, body fat, blood pressure, cardiorespiratory fitness, physical education lessons, log-linear analysis, multivariate correspondence analysis

## Abstract

**Simple Summary:**

In this study, we aimed to analyze the prevalence of positive effects of high-intensity interval training (HIIT) on body composition, cardiovascular parameters, and cardiorespiratory fitness among adolescents. We investigated 52 boys and 89 girls from a secondary school, separated into an experimental group (EG) with HIIT intervention and a control group (CG). The measured parameters were body fat % (BFP), resting systolic blood pressure (SBP), diastolic blood pressure (DBP), and fitness index (FI). The results indicate that positive HIIT-induced changes in SBP, DBP, and FI were most common among boys, especially those with low body mass index. Our study also revealed relationships between changes in FI and BFP and BP parameters. The effectiveness of HIIT was confirmed concerning the prevalence of the positive changes in measured parameters. We suggest that HIIT should be implemented in PE lessons, although there is a need to look for a more efficient method for girls.

**Abstract:**

Analysis of the interventions on cardiovascular disease risk factors focuses on quantitative changes, omitting assessment of positive effect frequency in individuals. The aim of this study was to assess the prevalence of positive effects of high-intensity interval training (HIIT) on body composition, cardiovascular parameters, and cardiorespiratory fitness among adolescents. A total of 52 boys and 89 girls from a secondary school were separated into an experimental group (EG) with HIIT and a control group (CG). Body fat % (BFP), resting systolic blood pressure (SBP), diastolic blood pressure (DBP), and fitness index (FI) changes were calculated. We assessed the influence and interaction of three factors: intervention (INT), sex (SEX), and body mass index (BMI_status_) on the ratio of individuals with and without positive changes. We used log-linear models for interactions and multivariate correspondence analysis (MCA). The results indicate that HIIT affects the prevalence of positive changes in SBP, DBP, and FI. Interactions between factors suggest boys with low BMI get more benefit from the intervention than girls. The MCA indicates a relationship between FI and BFP and between BP parameters. The effectiveness of HIIT was confirmed concerning the prevalence of the positive changes in measured parameters. We suggest that HIIT should be implemented in PE lessons, although there is a need to look for a more efficient method for girls.

## 1. Introduction

Obesity, high blood pressure (BP), and low cardiorespiratory fitness (CRF) in youth are associated the premature cardiovascular diseases and all-cause mortality in adulthood [1,2]. The literature has reported the prevalence of obesity in almost every fourth child and youth in the Western world; elevated BP (reflected in pre- and hypertension) in 11.2% of adolescents from developing countries; and low levels of CRF, which has declined over the past six decades [3,4,5].

There are strong associations between a high level of obesity, elevated BP, and lack of physical activity (PA) in children [6]. Epidemiological surveys demonstrated an increasing number of hypertension cases in youth linked to overweight/obesity in adolescents, particularly those who are not physically active [7]. A global growth in the prevalence of physical inactivity can be observed. About 80% of young people do not perform the minimum physical activity level recommended by the World Health Organization [8,9]. The same trend is observed in Poland [10]. One of the greatest global challenges is to improve the prevention and treatment of non-communicable diseases [11].

The effectiveness of the PA has been demonstrated in the regulation of body mass, resting blood pressure, also confirming its effectiveness in the prevention of obesity and hypertension [12,13]. The relevant setting to introduce PA seems to be physical education lessons [14]. Increasing the intensity of exercises in regular PE lessons at school seems to be the most accessible method [15]. Evidence suggests that implementing high-intensity exercises into physical education (PE) lessons positively correlates with improved blood pressure and body mass index [16,17,18,19]. High-intensity interval training (HIIT) seems to be the most appropriate method to increase exercise intensity in PE lessons. Its advantage is short intervention time with very intensive effort, which saves time but is enough to improve maximum oxygen uptake and affect cardiovascular parameters and body mass composition in adolescence [20,21,22,23,24,25]. As mentioned above, positive effects of HIIT intervention were observed in BP, body composition, and CRF [18,20,21,22,23,24,25]. However, some differences were observed in the relevant effects of sex and initial BMI [22,23,24,25]. This suggests differing effectiveness of HIIT intervention in various groups of adolescents. It remains unclear how the effectiveness of this type of intervention could differ due to sex and BMI, as well as the prevalence of positive effects on BP, body fat, and CRF.

Current studies on the effects of different interventions, including HIIT, on several aspects of biological condition and health of adolescents are primarily limited to the assessment of differences in measured variables (usually showing statistical significance of the difference in mean values) [26,27,28]. All studied groups included individuals who responded to experimental factors and individuals who did not respond. The question arises, what proportion of individuals experienced positive changes compared to those who experienced no such changes? The second question is: what factors moderate the mentioned relationship, and do these moderators interact with each other? The next question is: is there (and what type if yes) any correspondence between frequencies of individuals separated into different categories of factors (e.g., sex and body mass index)? To date, to our knowledge, there is a lack of an in-depth review of the effects of HIIT intervention on the prevalence of positive changes in adolescents, and there are, as yet, no answers to the questions posed above.

Therefore, the purpose of the present study was to assess the prevalence of positive changes in adolescents following high-intensity interval training (HIIT) implemented in physical education (PE) lessons in terms of body composition, cardiovascular parameters, and cardiorespiratory fitness according to sex and body mass index. Specifically, we aimed to (1) examine the potential interactions between factors that could affect positive changes in the mentioned parameters, (2) provide an overview of co-occurrences associated with the prevalence of positive changes in individuals following HIIT intervention, and (3) assess the structure and strength of similarities between categories.

## 2. Materials and Methods

### 2.1. Participants

The participants all participated in the same standard physical education program. The participants comprised six separate classes, of which three were randomly assigned to the experimental group (EG) and three to the control group (CG). Among 187 pupils from a secondary school, 141 subjects completed the study, comprising 52 boys (EG N = 31; CG N = 21; age, 16.24 (±0.34) years; body height, 176.74 (±6.07) cm; body mass, 65.42 (±12.51) kg) and 89 girls (EC N = 42; CG N = 47; age, 16.12 (±0.42) years; body height 164.38 (±6.54) cm; body mass 56.71 (±10.23) kg). Among the 46 excluded subjects, 10 were excluded due to medical contradiction, 17 were excluded from participating in additional sports training, and 19 were excluded during the intervention due to absence in physical education classes. There were no adverse effects observed. All participants were volunteers. A detailed description of the participants is presented in Table 1.

### 2.2. Procedures

The measurements were taken before and after the 10-week intervention on one day from 8:00 a.m. to 1:00 p.m. Participants were asked to excrete, avoid physical activity and excessive drinking of liquids, and keep their typical morning patterns directly before measurement.

### 2.3. Anthropometric Measurements

Body height was measured with an accuracy of 0.1 cm using anthropometers (GPM Anthropological Instruments, DKSH Ltd., Zurich, Switzerland). Bodyweight and body fat percentage (BF%) were measured using an InBody230 body composition analyzer (InBody Co. Ltd., Cerritos, CA, USA). This tool is characterized by very high reliability in men and women, as indicated by high intraclass correlation coefficients for BF% (≥0.98), FM (≥0.98), and FFM (≥0.99) and low standard error of measurement [29]. BMI was calculated based on received body height and weight values. Obtained results were used to divide participants into three groups based on the following intervals: low category of BMI_status_ (BMI < 20), medium category of BMI_status_ (19.99 < BMI < 23.00), and high category of BMI_status_ (BMI > 22.99). Intervals were arbitrarily assumed regarding small numbers of individuals with very low or very high body mass index.

### 2.4. Fitness Index (FI) (Harvard Step Test)—Cardiorespiratory Fitness

The Harvard Step Test (HST) was used to evaluate aerobic capacity. The HST results of subjects allowed for calculation of the fitness index (FI) according to the following formula [30]: PEI = (100 × *L*)/(5.5 × *p*), where *L* = duration of the test in seconds, *L* < 300 s, and *p* = heart rate within 1.5 min after the subject stopped the test. The reliability of the HST is acceptable at an intraclass correlations coefficient (ICC) of 0.63 [31].

The subjects had to step up and down on a stool with a height of 41.3 cm with a constant pace determined by a metronome. The process starts with the subjects stepping onto and off the step box at 30 cycles per minute. The test duration is a maximum of 300 s, or when the subject rejects the test due to fatigue. Resting heart rate and changes in pulse during exercise and recovery were measured (Polar H1 heart rate monitors, Polar Electro; Kempele, Finland). Heart rate monitors recorded pulse at 5 s intervals, which was transmitted to a smartwatch.

### 2.5. Resting Blood Pressure Measurements

Blood pressure was measured by an Omron BP710 automatic blood pressure monitor. The subjects had to sit quietly for 10 min. Next, the measurements were taken three times, separated into 10 min intervals. The analyzed results are the means of the three measurements.

### 2.6. Intervention

The intervention lasted 10 weeks. Participants followed the HIIT intervention during the one PE lesson (45 min) per week. First, a 10 min warmup with jogging and stretching exercises conducted. Next, the HIIT intervention was performed and lasted 14 min, divided into three sessions based on Tabata protocol (20 s work/10 s rest) separated by a 1 min break. In the first session, participants performed: pushups, high knees; in the second session: dynamic lunges, spider crawling; in the third session: plank-to-pushups and side squeezes. All exercises were played on a screen to ensure that workout and rest were implemented accurately. After the HIIT intervention, stretching and breathing exercises were performed to calm down. The control group participated in a standard physical education program.

The participants’ heart rate was measured with a Polar H1 (Polar Electro, Kempele, Finland) and established the range of 75–80% HRmax (145–157 heartbeats/min) when performing HIIT. The Tanaka formula, HRmax = 208 − 0.7 × “age” (age = 16 years in this study), was used to verify the intensity of the workout. The subjects achieved an HR of 156.2 ± 17.8 bpm (CI 95%: 123.0–184.0).

### 2.7. Statistical Analysis

Descriptive statistics for continuous measurements (BMI, BFP, SBP, DBP, and FI) are presented as means and SDs, with a 95% CI, and were calculated for boys and girls from experimental and control groups separated into BMI_status_ intervals.

The associations between the free factors and four outcomes were assessed in subsequent steps. The following categories of the elements were accepted: intervention (INT): experimental group (EG) with HIIT program vs. control group (CG) with standard PE lessons; sex (SEX): boys (M) vs. girls (F); BMI level (BMI_status_): low category vs. medium category vs. high category. The outcomes were changes between post- and preintervention results, coded as positive (1) or lack of positive (0) changes). The changes were calculated as post- and preintervention differences. Positive change was defined as any change consistent with the direction defined as pro-health, including reducing body fat and blood pressure (lower postintervention result than preintervention measurement), as well as incrementing cardiorespiratory fitness (higher value of the postintervention development than preintervention measurement). A lack of positive change was defined as no difference or results opposite to abovementioned changes.

First, log-linear analysis was conducted to find the simplest model that fits the data concerning outcome. Log-linear analysis is a method for studying structural relationships between variables in a contingency table [32]. This method examines which variables interact and impact outcomes [33]. Considering the log-linear model, we assumed that the natural logarithm of the value of an expected quantity in the table of independence is a linear function of factors. In a two-way case, the unrestricted log-linear model has the following form [34]:

log *π_ij_* = constant + *u*_1(*i*)_ + *u*_2(*j*)_ + *u*_12(*ij*)_

where *π_ij_* denotes the probability for cell (*i*, *j*), and {*u*} has to be constrained to identify the model.

An optimally designed log-linear model allows for the best quantitative prediction considering the smallest possible number of interactions. Pearson’s χ2 and χ2 maximum likelihood statistics assess whether the expected quantities are significantly different from the observed quantities [35] in order to determine the order of interactions that must be included in the model. To consider which interactions of a given order should be included in the model, it is necessary to analyze partial and marginal dependence. Partial support informs the significance of the degree of interactions, assuming that all other effects of the same degree are included in the model. Marginal dependence reflects the influence of the exchange, provided that the model does not have any interactions in the same order. Marginal dependence can be verified using the *χ*^2^ test of marginal interdependence [35]. Finally, we used Pearson’s *χ*^2^ to assess the model’s fit to the data. To examine the nature of effects (main and interactions), marginal quantity tables were calculated to observe quantity [36].

Next, multiple correspondence analysis (MCA) was performed to examine co-occurrences between categories of factors and variables. The analysis base was a matrix of individual results in the Burt table. Correspondence analysis is an exploratory technique often used to take an in-depth look at the results obtained from chi-square or log-linear analysis. Correspondence analysis applies to a two-way (or more) crosstabulation that summarizes the distribution of perceived categories of obtained variables in different groups (factors). MCA aims to reduce multidimensional space to more diminutive synthetic dimensions (mainly two main dimensions), which represent original data in the best way [37]. The measurement of dimensions is inertia, which can be compared to variance in ANOVA. It is possible to indicate variables (categories) strongly correlated with each dimension using squared cosine (*cos*^2^) to identify the nature of the synthetic dimension [38]. The result is a graph that plots data, visually showing the outcome of two or more data points.

The last step was to use cluster analysis (CIA) to graphically present the structure and similarities between categories linked together. In cluster analysis, we used Ward’s method of linkage and Euclidean distances. The raw data were coordinates (row and column profiles) obtained from MCA. Grouping a set of objects in ClA can be conducted so that items in the same group are more similar than those in other groups [35]. The result is a dendrogram that visually presents similarities and dissimilarities between associations.

The significance level was set at *α* = 0.05. Statistica V.13.0 statistical package (Tibco, 2020, Cracow, Poland) was used to analyze the study data.

## 3. Results

Descriptive statistics of the baseline values of measured parameters of the individuals in factor categories are presented in Table 1. Given that this work focused on qualitative rather than quantitative measured parameters, there was no calculation of statistics assessing differences between groups of adolescents.

The numbers of individuals who experienced positive or no positive changes (post-pre) in outcome after intervention are presented in Table 2. These frequencies were the starting point for building the Burt table used in correspondence analysis.

Due to the small number of respondents in relation to the total number of categories of three factors (seven categories), too many empty classes were created, and a complete analysis of the interactions between all factors was impossible. Therefore, we decided to conduct analyses for two-factor designs (two factors included in the model simultaneously). The following models were tested: INT*SEX→DV and INT*BMI_status_ →DV. Considering that the dependent variables are changes involving HIIT intervention, the SEX*BMI→DV model was omitted in case individuals from the experimental group and control group would be mixed up in categories of SEX and BMI_status_ factors, which would not make sense in terms of the purposes of this paper.

At the onset of the log-linear analysis, we determined the specification of the models, specifically the order of interactions. The test results of interactions are presented in Table 3. 

Regarding the information contained in Table 3, in both models (INT*SEX and HIIT*BMI_status_), apart from the main components, second-order interactions (at most) should be included. Third-order and higher interactions were not statistically significant. Partial and marginal tests were used to assess which interactions should be included in the model (Table 4).

The essence of the method is the assessment of the relevance of the interactions, which confirms that the effect of one causal variable on an outcome depends on the state of a second causal variable. An optimally designed log-linear model allows for the best quantitative prediction considering the smallest possible number of interactions.

Focusing only on interactions, the results of partial and marginal tests (Table 4) indicated the need to include two second-order interactions in the INT*SEX model: 41 and 61. Therefore, the strongest and most statistically significant influence of the HIIT intervention on SBP and FI was confirmed. In particular, there were more often positive changes in SBP and FI in the intervention group than in the control group. There was no interaction between INT and SEX. The impact of HIIT intervention on the prevalence of positive and lack of positive changes in SBP and FI was equal in boys and girls.

The same partial and marginal tests for the INT*BMI_status_ model indicated the need to include three interactions apart from main components: 41, 61, and 52. Interpretation of the results is similar to that of INT and SEX factors, with a small difference. We observed an interaction between BMI_status_ and DBP 52 The role of body-mass-to-height proportions was significant and induced positive changes in SBP. Detailed inspection of the marginal results indicated the highest rate of change in the low BMI_status_ category (the ratio of positive-to-lack of changes was 1.5 to 1), whereas the lowest for the high BMI_status_ category (the ratio of positive-to-lack of changes was 1 to 1).

The values of maximum likelihood statistics (L2), Pearson’s chi-squared statistics (*χ*^2^), and statistical non-significant p-values for both models (L2 = 46.64, *p* = 0.809, *χ*^2^= 45.47, *p* = 0.841; L2 = 53.52, *p* = 0.996, *χ*^2^ = 53.57, *p* = 0.996; respectively) confirmed models were well designed for the empirical data.

Implementation of the log-linear analysis allowed for a more detailed description of the interactions between the categories of factors and the amount of the positive and lack of positive changes after the intervention. Log-linear analysis was performed with a simple independence chi-squared test, which represents a more powerful tool to study dependence between qualitative data than an assessment based only on probability value [39].

**Figure 1:**
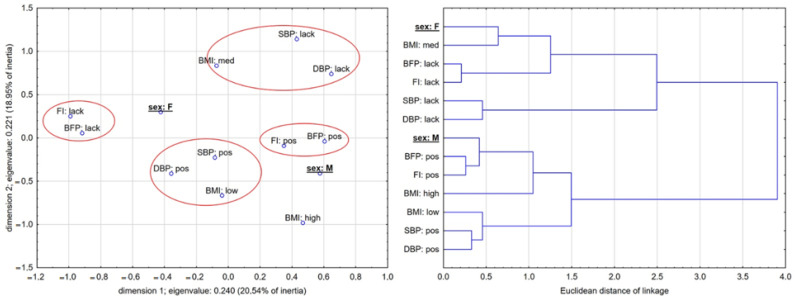
Results of intervention effects on the co-occurrence of changes in independent variables concerning sex and BMI_status_. Taxonomical dendrogram illustrates similarities and distances (strength) of linkages.

**Figure 2:**
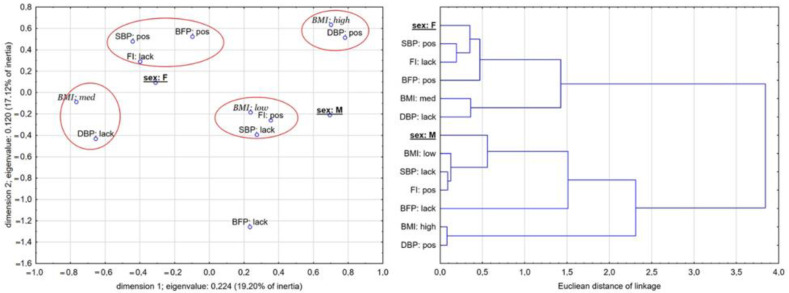
Co-occurrence of the changes in independent variables in the control group concerning sex and BMI_status_. Taxonomical dendrogram illustrates similarities and distances (strength) of linkages.

The results of MCA, shown in Figure 1 and Figure 2, present a 13-dimensional space of relationships reduced to two dimensions, the quality of presentation of which is acceptable. A projection of all variable associations in two-dimensional space describes over 40% of the total inertia (a measure of dispersion in categorical data that can be compared to variance for quantitative data) in the experimental group (first dimension showed 20,54% inertia, and the second dimension showed 18,95% inertia) and over 36% in the control group (19.20% and 17.12, respectively) (Figure 1 and Figure 2). Table 5 shows marginal quantities concerning observed quantities in both models for effects of interactions.

A comparison of marginal quantity to observed quantity in the INT*SEX model for SBP and FI confirmed more frequent positive changes in the experimental group compared to the control group (in proportions of 3.5:1 for SBP and 2.5:1 for FI). In the case of the HIIT*BMI_status_ model, proportions of positive changes in SBP and FI in EG compared to CG were similar to those mentioned above. In the case of BMI_status_, which interacts with DBP, for low-BMI_status_ and high-BMI_status_ categories of individuals, the ratio of positive changes to lack of positive changes was 1.5:1, whereas in medium-BMI_status_, the opposite was true.

Figure 1 and Figure 2 (EG and CG, respectively) present a clear separation between boys and girls (in both EG and CG) in the co-occurrence of changes in measured parameters. This indicates that SEX differentiation corresponds between categories of factors and variables independently of the intervention program. However, the picture of associations is quite different between EG and CG. In EG, the HIIT intervention response was higher in the male group. Girls more often exhibited a lack of changes in FI and BFP. Those categories of variables were very closely related. Boys from the low category of BMI_status_ more often exhibited positive changes in cardiovascular parameters. The structure and strength of similarities (based on distances between categories) are shown in dendrogram drawn using Ward’s method and Euclidean linkage distances. The FI and BFP categories were strongly linked, with the closest connection among all variables. This confirms that improvement in cardiorespiratory fitness went side by side with improvement (decrease) in the percentage of body fat. Distances between categories in a male cluster were larger than those in female clusters.

In CG, there were no clear connections, although categories of changes in boys and girls were similarly separated (Figure 2). Associations in the boy group were stronger, with closer correspondences than those in the girl group. Girls more often noted positive changes in SBP and BFP, which were closely connected with to a lack of changes in FI. Girls from the medium category of BMI_status_ more often noted a lack of DBP changes. Boys from the high category of BMI_status_ more often exhibited positive changes in DBP. Boys in the low category of BMI_status_ exhibited positive changes in FI, which corresponded with a lack of changes in SBP. The taxonomical dendrogram shows a more tight but less coherent structure than that of EG. Distances between categories of the variables in boys were shorter than those in girls. The closest linkage was noted for positive changes in boys from the high category of BMI_status_ and in boys from the low category of BMI_status_ for the interrelationship between lack of changes in SBP and positive changes in FI.

## 4. Discussion

The main findings were that 10-week HIIT implemented in PE lessons improved resting blood pressure and cardiorespiratory fitness and slightly reduced body fat, which was reflected in the prevalence of positive changes in EG compared to CG. Sex moderated the impact of HIIT in such a way that positive changes were more often noted in boys than in girls. The factor of BMI_status_ interacted with HIIT differently in boys and girls. The most positive changes were observed in boys from the low category of BMI_status_. In contrast, girls from the medium category of BMI_status_ exhibited the least positive changes. Secondly, a co-occurrence between some categories of variables was observed. Positive changes in FI were strongly related to positive changes in BFP, although mainly in boys, whereas the same co-occurrence related to FI and BFP but regarding a lack of positive changes was observed in girls.

Log-linear analysis allowed us to look deeper into the relationship between two factors potentially moderating INT effects. This is a unique approach compared to the use of simple independence chi-squared tests, which is mostly presented in the literature. Therefore, it is difficult to compare own results with those reported by others.

HIIT intervention has a broad influence on body composition and physical fitness. Our previous study [23] reported a significant decrease in mean values of body weight and BFP in response to HIIT implemented in physical education lessons. This effect was observed only in overweight subjects. Moreover, improvement in aerobic capacity was also observed. However, this effect was observed only among boys, which suggests sex as a factor differentiating HIIT effects. These results are convergent with the observations of other authors. In similar settings (PE lessons), Bogataj et al. [40] reported a positive impact of HIIT concerning body composition, with simultaneous improvement in physical fitness among obese girls. However, this effect was supported by additional nutrition intervention.

On the other hand, HIIT intervention effectively reduced waist and abdominal circumference directly associated with body fat [41]. Additionally, HIIT intervention is effective in cardiovascular improvement due to observation concern reduction of endothelial damage, which precedes atherosclerosis. A similar observation was noted by Tjonna et al. [42] independent of sex. Moreover, these authors noted improvement in blood pressure supported by improvement in metabolic parameters and body composition. In terms of cardiovascular status, a positive change in blood pressure was also observed in a study by Farah et al. [43].

Similarly to a previously cited study, a decrease in body weight was observed, with a simultaneous reduction in systolic and diastolic blood pressure among adolescents after HIIT. Sex is a factor that may affect HIIT effects. The literature often meets the approach with no respect to this factor, which may cause differentiative HIIT effects, as our observation confirms. This is supported by the results presented by Martinez-Vizcaino et al. [44]. Among boys, decreased body fat with free fat mass was noted. Reduce body fat was reported in girls, as well as a reduction in cardiometabolic risk through improvement in the blood lipid profile.

The observation mentioned above indicates simultaneous changes in body composition, cardiovascular parameters, and physical fitness in response to HIIT contributes evidence for interactions between these factors concerning sex and BMI_status_. Moreover, accessed studies analyze the changes quantitatively. There is no analysis concerning the prevalence of HIIT effects in studied subjects. To our knowledge, our current research is the first with to employ this approach.

Contrary to log-linear analysis, conducted to identify interactions between factors affecting a single outcome, MCA was used to assess multidimensional associations between all categories of characteristics and outcomes. A clear pattern of correspondence between categories was discovered in EG. HIIT involved parallel changes in FI and BFP, but SEX moderated that co-occurrence. Boys were closely related to positive changes, but girls lacked positive changes. An interaction effect of INT and BMI_status_ was discovered as close a relationship between positive changes in SBP and DBP in boys from the low BMI_status_ category. Other interaction effects were associations of the lack of positive changes in resting BP parameters in girls, mainly from the medium of BMI_status_ category. There was no clear pattern in the control group participating in a standard PE program. Changes were multidirectional and independent of SEX and BMI_status_ factors.

The literature presents results of the exercise training influence on the cardiovascular risk factor and cardiorespiratory fitness [45,46,47]. Many results revealed changes in body fat, cardiovascular parameters, or cardiorespiratory fitness separately and didn’t link associations in changes. Delgado-Floody et al. [28] first evaluated blood pressure changes and cardiorespiratory fitness improvements. No relationships were observed when testing the association between potential improvements in cardiorespiratory fitness and blood pressure improvements after the intervention. The same study showed decreases in percentage body fat (Δ−1.38%) in the experimental group, as well as SBP decreases (Δ−8.70 mmHg). Our results are slightly different, showing parallel changes in FI and BFP (similar to observations by Delgado-Floody [28]) but no relationship between BFP and BP (nor SBP and DBP). In our study, the relationship between changes in SBP and DBP was quite different in boys and girls.

Interestingly, boys from the low category of BMI_status_ benefited more from the intervention than others. It is worth noting that a reduction in systolic BP is related to a 7% decrease in cardiovascular disease risk [48]. Similarly, positive changes in CRF linked to BFP can be associated with health and a decrement of 15% in risk of cardiovascular disease in healthy boys, as documented by Kodama et al. [49].

Sex differences in positive changes between males and females in BP and CRF were observed in previous studies. Burgomaster et al. [50] revealed no change in VO_2_ max after HIIT, similar to earlier data [51] in men completing sprint training for eight weeks. In contrast, females exhibited changes in cardiorespiratory fitness [52]. Our results are contrary in the case of a frequency of girls compared to boys exhibiting no positive changes in either CRF or BFP and in both cardiovascular functions. Related results were obtained by Astorino et al. [53] concerning CRF and BP

The mechanism explaining positive changes in CRF, together with BFP in boys and opposed results in girls, as well as changes in resting blood pressure, is beyond the scope of this study. However, such a mechanism could be supposed to be associations with cardiac functions or O_2_ pulse, different in both sexes. It is difficult to compare our own results with those of others because no studies, to our best knowledge, have examined differences in proportions of individuals with positive or a lack of positive changes after HIIT intervention. What’s more, in our work, we presented co-occurrences between categories of factors and outcomes compared to multidimensional associations in quantitative data. No such analyses have been conducted until now.

Practical application refers to the recommendation to implement HIIT in PE lessons. We reported that regular 14 min intensive exercises (i.e., Tabata protocol) reduce health risk, particularly in boys (more prevalent changes), improving fitness and decreasing body fat and resting blood pressure. Measures related to the program were collected once a week.

A limitation of this study is the lack of prepubertal and peripubertal groups. Although studied groups of boys and girls were homogeneous in terms of age, adolescents who have finished their pubertal period could be at an advantage. Sexual maturation can significantly affect metabolic outcomes, so considering the effect of puberty on metabolism is vital for the validity of the results [19]. Controlling for sexual maturation (e.g., calculating maturity offset) would be a good solution. The second limitation was the lack of nutritional aspects, including investigating the effectiveness of interval protocols included in PE classes. Our study was limited to a once per week for a 10-week intervention during the school term. These conditions were forced by a need to simultaneously implement a standard PE program. Moreover, some limitations could be identified in a duration (14-min) intervention for one 45 min lesson. There was a need to conduct a warmup before HIIT and a cooldown after. On the other hand, due to possible side effects associated with the high-intensity effort with a higher volume of intervention, such an intervention could increase the risk of some side effects adolescents’ aversion to participating in the study. There is a need for follow-up studies to assess the durability of changes. Cardiorespiratory fitness maximal oxygen uptake (VO_2_ max) should be measured more objectively than can be achieved with the Harvard step-test, which has some limitations and does not assess CRF very precisely.

## 5. Conclusions

Our results bolster the importance of screening the effects of intervention in PE lessons in terms of percent of body fat, cardiovascular parameters, and cardiorespiratory fitness (which all are cardiovascular disease risk factors) not only as quantitative changes but also as the frequency of adolescents who benefit greatly from HIIT intervention comparing to peers participating in a standard PE program.

Positive changes are dependent on sex. Boys more often gained from intervention programs than girls, suggesting boys are more sensitive to HIIT influence. Interaction between BMI_status_ factor and intervention was observed within the male group. Boys with low BMI may benefit the most from HIIT intervention. Close associations were also revealed between positive changes in measured parameters: FI, BFP, and BP. This confirms the broad effect of HIIT as a valuable and effective tool to implement in PE lessons. However, there is a need to look for more efficient methods for girls, among whom fewer positive changes were noted. 

The usefulness of multidimensional methods was confirmed. Log-linear models were efficient in investigating interactions between factors and outcomes. After applying MCA, the multidimensional space of categorical data may be efficiently reduced to less-dimensional space (mostly two-dimensional). Moreover, it is possible to study multidimensional relationships, called co-occurrence between categories of factors. Calculating distances using cluster analysis allowed us to assess the structure of linkages and similarities between groups of factors and dependent variable categories. The information obtained in this way might be a valuable clue to take practical action to decrease or increase the severity of the studied phenomena.

The conclusions mentioned above could be helpful for scientists studying the effects of interventions on cardiovascular disease risk factors. Interpreting the results from a public health point of view, inherent associations between the investigated variables can be a road map for authorities to plan their policies more efficiently.

Subsequent studies should focus on the prevalence of responders and non-responders in terms of body fat, blood pressure, and cardiorespiratory fitness after high-intensity interval training in adolescents.

## Figures and Tables

**Table 1:** Descriptive statistics of dependent variables (body fat percentage (BFP), resting systolic and diastolic blood pressure (SBP, DBP), and fitness index (FI)) in categories of factors (intervention (INT), sex (SEX), and body mass index intervals (BMI_status_).

Factors				Outcomes			
INT	SEX	BMI_status_		BFP	SBP	DBP	FI
		Category	Mean ± SD 95%CI	Mean ± SD 95%CI	Mean ± SD 95%CI	Mean ± SD 95%CI	Mean ± SD 95%CI
EG	M	L	18.36 ± 1.35 17.61–19.11	11.53 ± 2.93 9.90–13.15	121.40 ± 14.96 113.11–129.68	76.46 ± 5.93 73.17–79.75	44.81 ± 4.30 42.43–47.19
		M	21.00 ± 0.98 20.33–21.66	15.87 ± 4.60 12.77–18.96	123.63 ± 13.33 114.67–132.59	69.90 ± 6.93 65.25–74.56	45.14 ± 2.30 43.59–46.69
		H	28.26 ± 3.80 23.53–32.98	27.62 ± 6.67 19.32–35.91	128.40 ± 4.21 123.16–133.63	76.20 ± 9.49 64.40–87.99	42.65 ± 2.80 39.16–46.14
	F	L	18.62 ± 1.01 18.08–19.16	23.90 ± 2.88 22.37–25.44	116.87 ± 10.06 111.51–122.23	73.43 ± 8.81 68.73–78.13	42.28 ± 2.60 40.90–43.67
		M	21.47 ± 0.96 21.06–21.89	28.12 ± 5.32 25.82–30.43	116.69 ± 7.87 113.28–120.10	70.26 ± 6.46 67.46–73.05	44.32 ± 5.35 42.01–46.64
		H	23.97 ± 0.81 21.95–25.99	30.96 ± 3.00 23.51–38.42	117.01 ± 6.24 101.48–132.51	73.00 ± 6.08 57.88–88.11	42.64 ± 7.19 24.77–60.51
CG	M	L	18.43 ± 0.98 17.67–19.19	11.75 ± 3.17 9.31–14.19	116.00 ± 8.30 109.61–122.38	74.77 ± 5.51 70.53–79.01	43.21 ± 2.89 40.99–45.43
		M	21.30 ± 0.86 20.58–22.02	13.43 ± 3.14 10.80–16.06	122.25 ± 11.20 112.87–131.62	77.12 ± 5.74 72.32–81.92	45.36 ± 3.59 42.35–48.36
		H	25.55 ± 2.58 21.43–29.66	24.27 ± 10.00 8.34–40.20	128.75 ± 3.30 123.49–134.00	79.25 ± 10.71 62.19–96.30	41.93 ± 2.96 37.21–46.66
	F	L	18.41 ± 0.95 17.97–18.84	24.55 ± 3.86 22.79–26.31	113.28 ± 6.39 110.37–116.19	70.19 ± 5.52 67.67–72.70	44.08 ± 3.63 42.43–45.73
		M	21.57 ± 1.05 20.98–22.15	29.40 ± 3.31 27.56–31.24	116.46 ± 9.22 111.35–121.57	68.60 ± 7.64 64.36–72.83	44.44 ± 3.49 42.50–46.37
		H	26.21 ± 4.45 23.22–29.20	35.93 ± 5.83 32.01–39.85	117.72 ± 9.76 111.16–124.28	72.27 ± 9.33 65.99–78.54	45.79 ± 5.13 42.34–49.24

INT-intervention factor, categories: EG-experimental group, CG-control group; SEX-sex factor, categories: M-male, F-female; BMI_status_-BMI factor, categories: L-low, M-medium, H-high; BFP-percentage of body fat; SBP-systolic blood pressure, DBP-diastolic blood pressure, FI-fitness index.

**Table 2:** Numbers and frequencies of individuals in categories of each factor (INT, SEX, and BMI_status_) with positive (+) and no positive (−) changes after intervention in measured parameters (BFP, SBP, DBP, and FI).

FACTOR		DV							
		BFP		SBP		DBP		FI	
		−	+	−	+o	−	+	−	+
		N (%)	N (%)	N (%)	N (%)	N (%)	N (%)	N (%)	N (%)
INT	EG	29 (39.73)	44 (60.27)	12 (16.44)	61 (83.56)	26 (35.62)	47 (64.38)	19 (26.03)	54 (73.97)
	CG	20 (29.41)	48 (70.59)	42 (61.76)	26 (38.24)	20 (29.41)	48 (70.59)	32 (47.06)	36 (52.94)
SEX	M	18 (34.62)	31 (65.38)	18 (34.62)	34 (65.38)	21 (40.38)	31 (59.62)	16 (30.77)	36 (69.23)
	F	31 (34.83)	58 (65.17)	36 (40.45)	53 (59.55)	42 (47.19)	47 (52.81)	35 (39.33)	54 (60.67)
BMI_status_	L	23 (37.70)	38 (62.30)	24 (39.34)	37 (60.66)	22 (36.07)	39 (63.93)	20 (32.79)	41 (67.21)
	M	20 (35.09)	37 (64.91)	19 (33.33)	38 (66.67)	32 (56.14)	25 (43.86)	21 (36.84)	36 (63.16)
	H	6 (26.09)	17 (73.91)	11 (47.83)	12 (52.17)	9 (39.13)	14 (60.87)	10 (43.48)	13 (56.52)

− lack of positive changes; + positive changes; INT-intervention factor, categories: EG-experimental group, CG-control group; SEX-sex factor, categories: M-male, F-female; BMI_status_-BMI factor, categories: L-low, M-medium, H-high; BFP-percentage of body fat; SBP-systolic blood pressure; DBP-diastolic blood pressure; FI-fitness index.

**Table 3:** Test results of interactions between factors (1–2) and dependent variables (BFP, SBP, DBP, and FI).

k-Factors	INT(1)*SEX(2)			INT(1)*BMI_status_(2)		
	df	*χ* ^2^	*p*	df	*χ* ^2^	*p*
1	6	53.30	0.0000	7	66.28	0.0000
2	15	43.00	0.0002	20	48.21	0.0004
BFP	20	22.39	0.3197	30	17.47	0.9666
SBP	15	10.60	0.7807	25	16.32	0.9049
DBP	6	3.12	0.7952	11	6.84	0.8116
FI	1	0.94	0.3317	2	0.26	0.8777

INT-intervention factor, BMI_status_-BMI factor, SEX-sex, BFP-percentage of body fat, SBP-systolic blood pressure, DBP-diastolic blood pressure, FI-fitness index.

**Table 4:** Results of the partial (*χ*^2^ part) and marginal (*χ*^2^ marg) tests between the factors (1*2) and dependent variables (BFP, SBP, DBP, and FI): main effects and interactions (only selected interactions are presented).

Effect	INT(1)*SEX(2)					INT(1)*BMI_status_(2)				
	df	*χ* ^2^_part_	*p*	*χ* ^2^_marg_	*p*	df	*χ* ^2^_part_	*p*	*χ* ^2^_marg_	*p*
1	1	0.14	0.7038			1	0.13	0.7160		
2	1	7.97	0.0047			2	15.01	0.0006		
3 (BFP)	1	10.80	0.0010			1	9.87	0.0017		
4 (SBP)	1	6.33	0.0118			1	5.79	0.0161		
5 (DBP)	1	1.30	0.2538			1	1.19	0.2750		
6 (FI)	1	8.87	0.0029			1	8.11	0.0044		
12	1	0.97	0.3240	1.57	0.2103	2	2.19	0.3341	2.81	0.2456
13	1	1.73	0.1888	1.25	0.2628	1	1.54	0.2144	1.12	0.2904
14	1	22.86	0.0000	25.27	0.0000	1	19.72	0.0000	22.94	0.0000
15	1	2.01	0.1561	4.13	0.0422	1	2.59	0.1075	3.78	0.0518
16	1	4.57	0.0324	5.47	0.0194	1	4.15	0.0416	4.99	0.0255
23	1	0.01	0.9345	0.02	0.8848	2	0.24	0.8862	0.21	0.8994
24	1	0.00	0.9840	0.23	0.6306	2	0.61	0.7388	1.44	0.4863
25	1	0.19	0.6599	0.41	0.5210	2	4.83	0.0894	3.91	0.1417
26	1	0.28	0.5971	0.56	0.4545	2	0.83	0.6610	1.03	0.5975
34	1	0.13	0.7204	0.01	0.9235	1	0.12	0.7284	0.00	0.9695
35	1	0.01	0.9210	0.05	0.8298	1	0.00	0.9568	0.03	0.8564
36	1	1.29	0.2560	0.83	0.3634	1	1.31	0.2530	0.85	0.3554
45	1	0.76	0.3829	2.45	0.1174	1	0.97	0.3254	2.28	0.1309
46	1	0.00	0.9882	0.84	0.3588	1	0.01	0.9293	0.85	0.3579
56	1	0.15	0.6955	0.57	0.4514	1	0.14	0.7073	0.55	0.4603

INT-intervention factor, BMI_status_-BMI factor, SEX-sex, BFP-percentage of body fat, SBP-systolic blood pressure, DBP-diastolic blood pressure, FI-fitness index.

**Table 5:** Table of marginal quantity concerning observed quantity in INT*SEX and INT-BMIstatus models for dependence: INT effect in SBP and INT effect in FI in the first model and INT effect in SBP, INT effect in FI, and BMIstatus effect in DBP in the second model.

	INT*SEX				INT*BMI_status_						
	INT*SBP		INT*FI		INT*SBP		INT*FI		BMI_status_*DBP		
	EG	CG	E	CG	EG	CG	EG	CG	L	M	H
0	20	50	27	40	24	54	31	44	30	40	17
1	69	34	62	44	73	38	66	48	47	33	22
all	89	84	89	84	97	92	97	92	77	73	39

INT-intervention factor, categories: EG-experimental group, CG-control group; SEX-sex factor, categories: B-boys, G-girls; BMI_status_-BMI factor, categories: L-low, M-medium, H-high; BFP-percentage of body fat; SBP-systolic blood pressure; DBP-diastolic blood pressure; FI-fitness index.

## Data Availability

The data presented in this study are available upon request from the corresponding author.
